# Eastern Cooperative Oncology Group, **β**2-microglobulin, hemoglobin, and lactate dehydrogenase can predict early grade ≥ 3 infection in patients with newly diagnosed multiple myeloma: A real-world multicenter study

**DOI:** 10.3389/fmicb.2023.1114972

**Published:** 2023-01-27

**Authors:** Xinyi Lu, Wenhua Liu, Lan Zhang, Xinyue Chen, Liping Yang, Qiong Yao, Jie Zhao, Shaolong He, Jia Wei, Weiwei Tian

**Affiliations:** ^1^Department of Hematology, Third Hospital of Shanxi Medical University, Shanxi Bethune Hospital, Shanxi Academy of Medical Sciences Tongji Shanxi Hospital, Taiyuan, Shanxi, China; ^2^Department of Hematology, Shanxi Provincial People’s Hospital, Taiyuan, Shanxi, China; ^3^Department of Hematology, First Hospital of Shanxi Medical University, Taiyuan, Shanxi, China; ^4^Sino-German Joint Oncological Research Laboratory, Shanxi Bethune Hospital, Shanxi Academy of Medical Sciences, Taiyuan, Shanxi, China; ^5^Department of Hematology, Tongji Hospital, Tongji Medical College, Huazhong University of Science and Technology, Wuhan, Hubei, China

**Keywords:** multiple myeloma, infection, predictive model, frail, Bortezomib

## Abstract

**Introduction:**

This research explored the clinical application of grade ≥ 3 infection predictive models for the newly diagnosed multiple myeloma (NDMM) population.

**Methods:**

It evaluated 306 patients with NDMM based on three different predictive models. The relationship between the grade ≥ 3 infection rates in NDMM and the scores was analyzed retrospectively. The cumulative incidence of early grade ≥ 3 infection was estimated using the Kaplan–Meier method and log-rank test to assess the statistical significance of the difference. To compare the predictive performance in the prediction of infection, the Receiver Operating Characteristic Curve (ROC) curve was used to show the area under the curve (AUC), and DeLong’s test was used to analyze the difference in AUC.

**Results:**

The incidence of grade ≥ 3 infection within the first 4 months of NDMM was 40.20%. Concerning the FIRST score (predictors: ECOG, β2-microglobulin, hemoglobin, and lactate dehydrogenase), GEM-PETHEMA score (predictors: albumin, male sex, ECOG, and non-IgA type MM), and Infection Risk model of Multiple Myeloma (IRMM) score (predictors: ECOG, serum β2-microglobulin, globulin, and hemoglobin), the probability of early grade ≥ 3 infection in the different groups showed statistically significant differences (low-risk vs. high-risk: 25.81% vs. 50.00%, *p* < 0.001; low-risk vs. moderate-risk vs. high-risk: 35.93% vs. 41.28% vs. 60.00%, *p*= 0.045; low-risk vs. moderate-risk vs. high-risk: 20.00% vs. 43.75% vs. 52.04%, *p* < 0.001). Statistical differences existed in the probability of early grade ≥ 3 infection among the different groups by the FIRST and IRMM scores but no statistical differences in the GEM-PETHEMA score (*p* < 0.001, *p*< 0.001, and *p* = 0.090, respectively). The FIRST score showed good discrimination and simple calculation with highest AUC. Further subgroup analysis showed that the FIRST score could still apply for patients treated with bortezomib-based regimen and frail patients.

**Discussion:**

Our findings indicate that the FIRST score (consisting of ECOG, β2-microglobulin, hemoglobin, and lactate dehydrogenase) is a simple and robust infection stratification tool for patients with NDMM and could be used in routine clinical work.

## 1. Introduction

In recent years, survival in multiple myeloma (MM) has improved significantly. However, no significant decrease has occurred in early mortality. Infection is a common early complication of newly diagnosed multiple myeloma (NDMM) and a major cause of early death. One study showed that 45% of early deaths within 6 months were due to infection (Augustson et al., 2005). Some studies have shown that infection was listed as a contributing factor to death (McQuilten et al., 2022). Furthermore, even if the early infection is not fatal, it frequently causes significant delays and dose reductions in subsequent treatment, raising the risk of treatment failure (Zahid et al., 2019). Consequently, detecting early infection is critical to reducing early mortality.

The scoring system can help determine a patient’s risk of infection during MM treatment, allowing risk-adaptive strategies to be implemented to prevent early infection. However, an accurate infection prediction necessitates time and massive data. If the current infection prediction model can categorize infection in the real world, constructing a new prediction model seems unnecessary. At present, three infection risk prediction models have been proposed. In 2018, the FIRST score (Dumontet et al., 2018) was proposed first to stratify the risk of early grade ≥ 3 infections in patients with NDMM. This score is based on Eastern Cooperative Oncology Group (ECOG) performance status and serum β2-microglobulin, lactate dehydrogenase, and hemoglobin levels to define high-risk and low-risk groups showing significantly different rates of grade ≥ 3 infection (24.0% vs. 7.0%, respectively; *p* < 0.001) in the first 4 months. In 2022, the GEM-PETHEMA score (Encinas et al., 2022) comprising serum albumin, ECOG, male sex, and MM type was established to facilitate the identification of three risk groups with different probabilities of severe infection within the first 4 months, using the data from GEM2005 > 65, GEM2005 < 65, and GEM2012 < 65. The authors found that the infection rates of low-risk, intermediate-risk, and high-risk patients were 8.2, 19.2, and 28.3%, respectively. The two models showed good effects in the prediction of NDMM ≥3 infections. However, in addition to uniform clinical trials, a robust infection risk stratification system should perform well in the real-world setting. Since all patients were recruited as part of the clinical trial, they all met the inclusion and exclusion criteria. In the real world, more patients have poor physical fitness. In addition, in China, more patients receive a bortezomib-based regimen. However, unlike, GEM-PETHEMA score, participants in the FIRST score mostly accepted a non-bortezomib regimen. Consequently, determining whether the FIRST score and GEM-PETHEMA score can be used in the real world is essential.

IRMM is an infection prediction model established in China using real-world data (Shang et al., 2022). This model uses performance status, hemoglobin, β2-microglobulin, and globulin to categorize patients into high-risk, moderate-risk, and low-risk groups, which has shown significantly different rates of early infection in the three cohorts (46.5% vs. 22.1% vs. 8.8%; *p* < 0.001). The results indicated that the IRMM model could predict early grade ≥ 3 infection. However, it has not been validated in the real world by other centers’ data. Validating IRMM scores with data from other facilities would also be logical.

Therefore, the purpose of this study was to determine which of these three prediction models can clearly recognize grade ≥ 3 infection in NDMM patients in real-world clinical practice, enabling clinicians to determine appropriate infection prevention strategies.

## 2. Materials and methods

### 2.1. Patient population and study design

**Figure 1:**
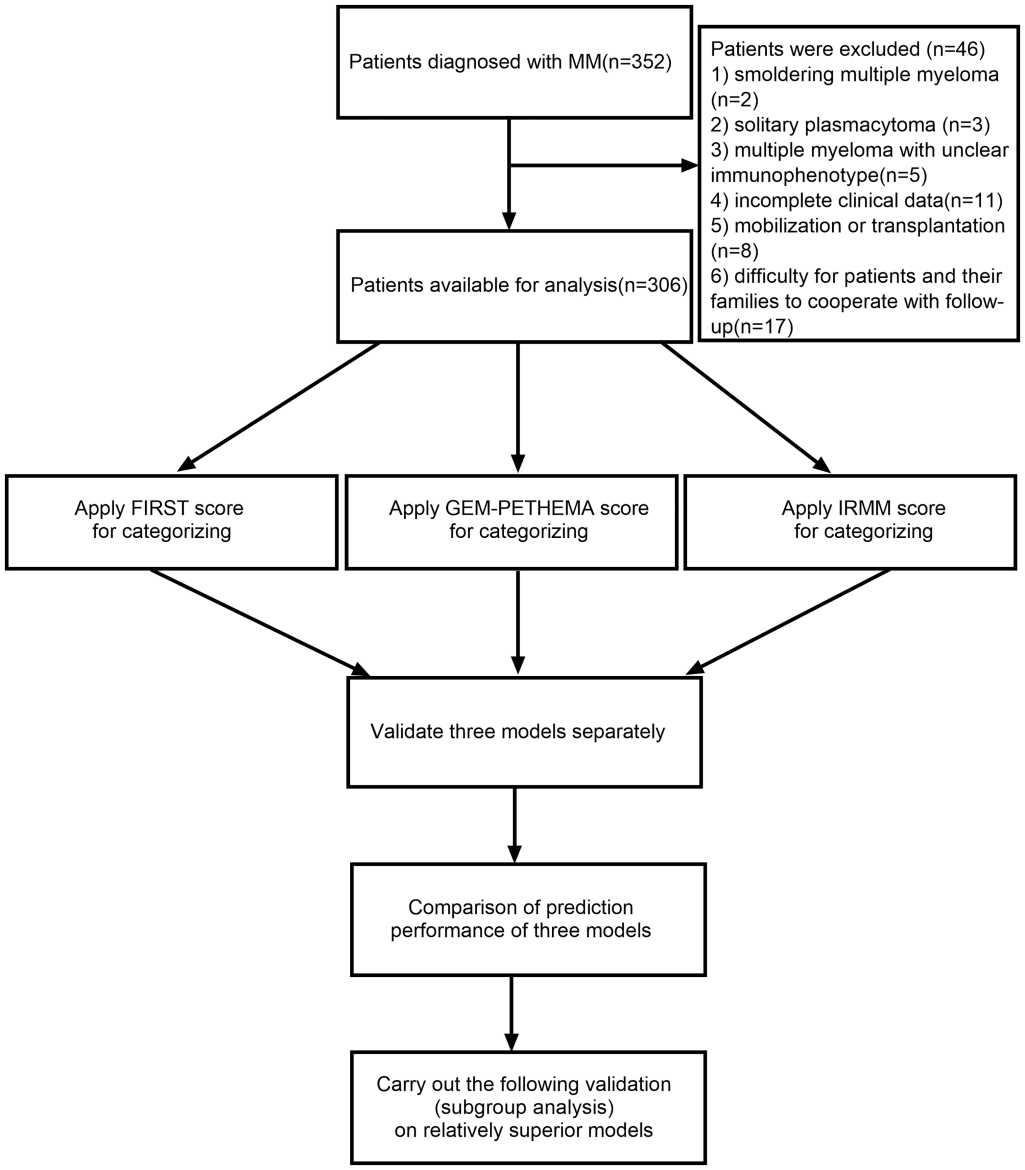
The flow diagram in this study. Using the International Myeloma Working Group (IMWG) criteria 352 patients were diagnosed with multiple myeloma (MM). The following reasons led to the exclusion of 46 patients: (1) smoldering MM (*n* = 2), (2) solitary plasmacytoma (*n* = 3), (3) MM with unclear immunophenotype (*n* = 5), (4) incomplete clinical data (*n* = 11), (5) mobilization or transplantation (*n* = 8), and (6) difficulty for patients or their families to cooperate with follow-up (*n* = 17). FIRST score was used for assignment and grouping: β2-MG ≤ 3 mg/l was assigned-2 points; ECOG < 1 was assigned-1 point; hemoglobin ≤ 110 g/l, ECOG ≥ 2, LDH ≥ 200 U/l was assigned 1 point; β2-MG ≥ 6 mg/l was assigned 2 points. We defined-3-1 point as low-risk and 2–5 points as high-risk. GEM-PETHEMA score was used for assignment and grouping: serum albumin ≤ 30 g/l, ECOG > 1, male, non-IgA MM was assigned a score of 1. We defined 0–2 point as low-risk, 3 points as moderate risk, and 4 points as high-risk. IRMM score was used for assignment and grouping: ECOG ≥ 2, β2-MG ≥ 6 mg/l assigned two points; hemoglobin was < 35 g/l of the lower limit of the normal range, and globulin ≥ 2.1 times the upper limit of the normal range assigned 1 point. We defined 0–1 point as low-risk, 2–3 points as moderate risk, and 4–6 points as high-risk. (In this study, the lower limit of the normal range for hemoglobin was 115 g/l and the upper limit of the normal range for globulin was 30 g/l.)

A total of 306 patients with NDMM in the Third Hospital of Shanxi Medical University, First Hospital of Shanxi Medical University, and Shanxi Provincial People’s Hospital from May 1, 2016, to April 30, 2022, were enrolled in this retrospective study. All patients were diagnosed with MM using the International Myeloma Working Group (IMWG) criteria (Rajkumar et al., 2014). The exclusion criteria were (1) smoldering MM, (2) solitary plasmacytoma, (3) MM with unclear immunophenotype, (4) incomplete clinical data, (5) mobilization or transplantation, and (6) difficulty for patients or their families to cooperate with follow-up. The flow diagram for this study is depicted in Figure 1. The institutional medical ethics committee provided its approval to the protocol (Ethical approval number YXLL-2022-111), and it was carried out in compliance with the Declaration of Helsinki.

### 2.2. Definitions

The clinical manifestations of the patient, typical imaging findings of infection, or the isolation of a microbial agent from peripheral blood or secretions in patients who also had concurrent clinical symptoms all served as the criteria for infection used in this study (Shang et al., 2022). The severity of infection was evaluated using the Common Terminology Criteria for Adverse Events (CTCAE) v5.0 published by the National Cancer Institute of the National Institutes of Health, United States Department of Health and Human Services. Infections classified as grade 3 require invasive intervention, such as intravenous antibiotics, antifungals, or antiviral therapy. Grade ≥ 3 infection consists of grade 3 infections, grade 4 infections (life-threatening consequences), and grade 5 infections (death). Early infection was considered as infection developing during the first 4 months of treatment.

Patients were identified as nonfrail or frail based on their age, Charlson Comorbidity Index (CCI), and ECOG performance status (Facon et al., 2020): ≤ 75 years old, CCCI ≤ 1, and ECOG = 0 was assigned 0 points; 76–80 years old, CCI > 1, and ECOG = 1 was assigned 1 point; and > 80 years old and ECOG ≥ 2 was assigned two points. We defined nonfrail as 0–1 points and frail as ≥ 2 points.

### 2.3. Collected parameters

Data were collected by studying medical records. The following factors were considered: age, gender, subtype, International Staging System (ISS) stage, Durie–Salmon (DS) stage, ECOG performance status, frailty assessment [the simplified frailty scale (Facon et al., 2020) was used to evaluate frailty], first-line treatment protocol (non-bortezomib-based or bortezomib-based), hemoglobin, platelets, white blood cells (WBC), serum β2-microglobulin (β2-MG), lactate dehydrogenase (LDH), serum albumin (ALB), serum globulin (GLB), infection status, severity of infection, classification of infection, sites of infection, and microbial species.

### 2.4. Predictive tools

The FIRST score, GEM-PETHEMA score, and IRMM score were employed to categorize enrollment populations. The values of the three predictive models and the grouping of risk levels are shown in Supplementary Table 1.

### 2.5. Statistical analysis

The data were analyzed using SPSS 26.0 statistical software, with count data expressed as frequencies and percentages (%), continuous data conforming to a normal distribution expressed as *x* ± s, and continuous data not conforming to a normal distribution expressed as medians (interquartile spacing). The continuous data were analyzed by analysis of variance, and the count data were analyzed by Chi-square test or Fisher exact probability method. The cumulative incidence of early grade ≥ 3 infection was estimated using the Kaplan–Meier method and log-rank test to assess the statistical significance of the difference. To compare the predictive performance in the prediction of infection, the ROC curve was used to show the area under the curve (AUC), and DeLong’s test was used to analyze the difference in AUC. Statistical significance was defined as a two-sided *p*-value of < 0.05.

## 3. Results

### 3.1. Patient characteristics and baseline comparison

A total of 306 patients with a median age of 64 years (IQR 57–71 years) were enrolled in the trial, comprising 172 men and 134 women. They included 144 IgG cases (47.06%), 70 IgA cases (22.88%), 65 light-chain cases (21.24%), 20 IgD cases (6.54%), 5 biclonal cases (1.63%), 1 IgM case (0.33%), and 1 oligosecretory case (0.33%). There were 12 cases (3.92%) of DS stage I, 29 cases (9.48%) of stage II, and 265 cases (86.60%) of stage III. There were 29 (9.48%) cases of ISS stage I, 113 (36.93%) cases of stage II, and 164 (53.59%) cases of stage III; 191 (62.42%) patients were in the frail group, and 115 (37.58%) were in the nonfrail group; 269 (87.91%) patients were treated with the bortezomib-based regimen, and 37 (12.09%) were treated with the non-bortezomib-based regimen. Patients receiving bortezomib-based therapy were routinely given drugs such as acyclovir or valacyclovir to prevent viral infections, but all patients were not used preventive antibacterial or fungal drugs. Table 1 illustrates the baseline characteristics of patients with NDMM.

**Table 1:** Baseline clinical characteristics of the patients with newly diagnosed multiple myeloma (NDMM).

Variables	Number of patients (%)/M ± SD
**Age (years)**	
≥ 65	152 (49.67%)
Sex Male	172 (56.21%)
MM subtype	
IgA	70 (22.88%)
Non-IgA	236 (77.12%)
ISS stage	
I–II	142 (46.41%)
III	164 (53.59%)
**DS stage**	
I–II	41 (13.40%)
III	265 (86.60%)
**ECOG**	
0	30 (9.80%)
1	101 (33.01%)
2–4	175 (57.19%)
Frailty assessment	
Frail	191 (62.42%)
Non-frail	115 (37.58%)
**Treatment protocol**	
Bortezomib-based	269 (87.91%)
Non-bortezomib-based	37 (12.09%)
Hemoglobin (g/L)	92.02 ± 24.04
Platelet (×10^9^/L)	169.61 ± 85.21
WBC (×10^9^/L)	5.21 ± 2.86
Serum β2-microglobulin (mg/L)	7.36 ± 6.24
Lactate dehydrogenase (U/L)	189.83 ± 123.01
Albumin (g/L)	31.82 ± 7.20
Globulin (g/L)	48.83 ± 29.13

NDMM, newly diagnosed multiple myeloma; MM, multiple myeloma; IgA immunoglobulin A; ISS stage, international staging system stage; DS stage, Durie-Salmon stage; ECOG, Eastern Cooperative Oncology Group performance status, Bortezomib-based Bortezomib + Cyclophosphamide + Dexamethasone (VCD), Bortezomib + Lenalidomide + Dexamethasone (VRD), Bortezomib + Pomalidomide + Dexamethasone (VPD), Bortezomib + Thalidomide + Dexamethasone (VTD), Bortezomib + Dexamethasone(VD), Bortezomib (V), Bortezomib + Thalidomide + Dexamethasone + Cyclophosphamide (VTDC), Bortezomib + Dexamethasone + Etoposide + Cyclophosphamide + Cisplatin (VDECP), etc. Non-bortezomib-based Lenalidomide + Cyclophosphamide + Dexamethasone (RCD), Thalidomide + Cyclophosphamide + Dexamethasone (TCD), Ixazomib + Lenalidomide + Dexamethasone (IRD), Ixazomib + cyclophosphamide + Dexamethasone (ICD), Lenalidomide + Dexamethasone (RD), Ixazomib + Dexamethasone (ID), Thalidomide + Dexamethasone (TD), Doxorubicin + Vincristine + Dexamethasone (DVD), Lenalidomide (R), etc. WBC, white blood cells; *M* ± SD, mean ± standard deviation.

### 3.2. Early infection events

**Figure 2:**
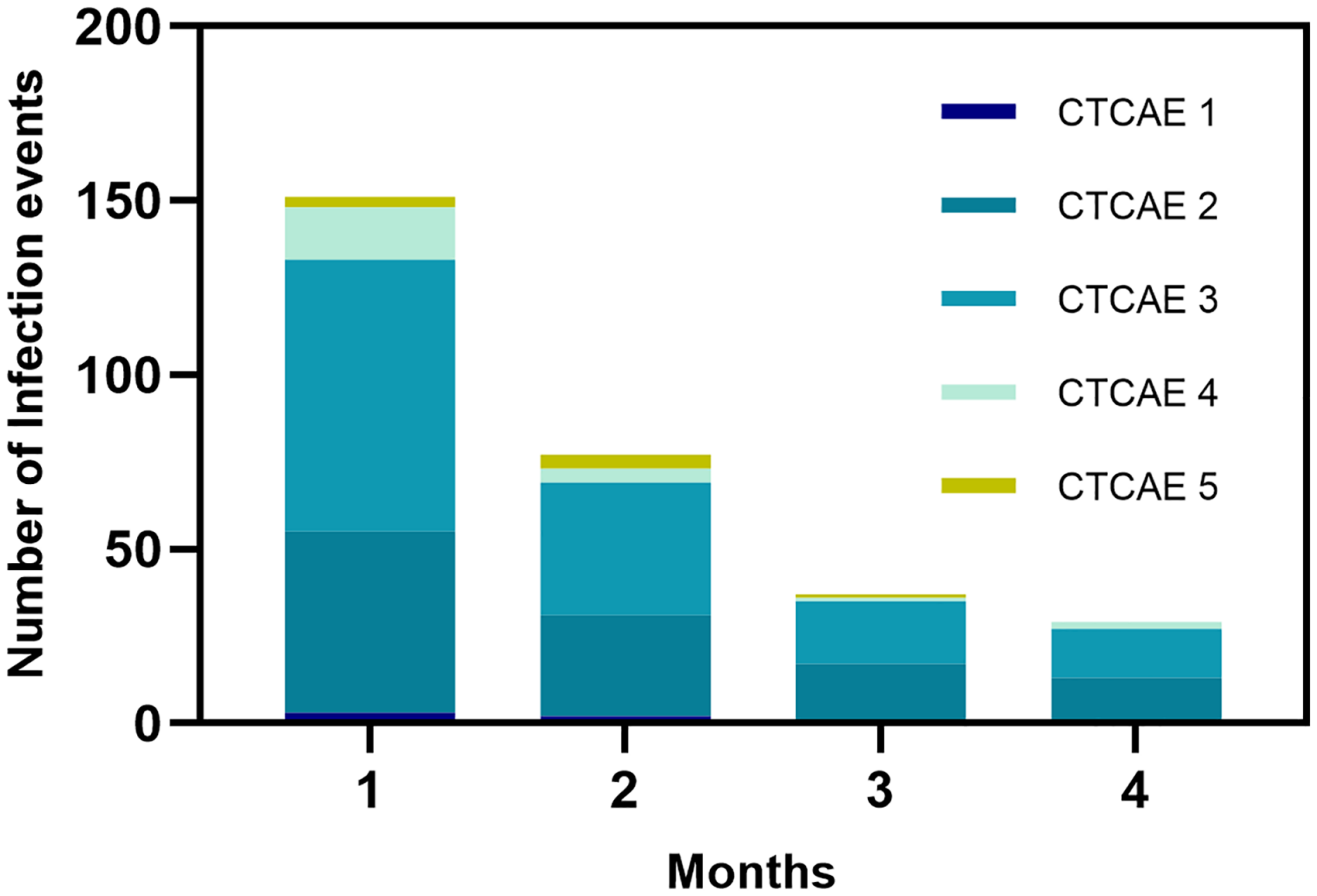
Infection events and grading within the first 4 months. The severity of infection was evaluated using the Common Terminology Criteria for Adverse Events v5.0 (CTCAE) published by the National Cancer Institute of the National Institutes of Health, United States Department of Health and Human Services.

Of the 306 patients with NDMM, 202 (66.01%) experienced 294 early infections (within 4 months). Of them, 75 patients had two or more infection events, and 151 (51.36%) infections took place in the first month (Figure 2). CTCAE grade 3 or above was observed in 178 (60.54%) of the infectious episodes of 123 patients (40.20%; Supplementary Table 2). There were no significant differences in infections between different induction treatment (Supplementary Table 3). The median time to onset of infection among the 178 cases of grade ≥ 3 infection was 0.77 months. 15 died, 8 (53.33%) died of infection, 4 (26.67%) died of myeloma progression,2 (13.33%) died of organ failure, and 1 (6.67%) died of unknown reason.

The most common sites of early grade ≥ 3 infections were the lower respiratory tract (132, 44.90%), upper respiratory tract (88, 29.93%), and gastrointestinal tract (27, 9.18%). There were 59 bacterial infections, 20 viral infections, and 9 fungal infections (3 cases were combined bacterial and fungal infections or combined bacterial and viral infections). *Escherichia coli* was the most common Gram-negative organism, *Enterococcus faecalis* was the most common Gram-positive organism, and *Candida albicans* was the most common fungus. Classification and constituent ratios (%) of pathogens in patients with NDMM are shown in Supplementary Table 4.

### 3.3. Evaluation of FIRST score

Patients were divided based on the FIRST score into a low-risk group (124, 40.52%) and a high-risk group (182, 59.48%). The difference in grade ≥ 3 infection between the two groups was statistically significant (*p* < 0.001), and the probability of early grade ≥ 3 infection was significantly higher in the high-risk group than in the low-risk group (50.00% vs. 25.81%, *χ*^2^ = 17.958, *p* < 0.001; Table 2). The OR (95% CI) for the high-risk group vs. low-risk group was 2.875 (1.750–4.722). The clinical characteristics of MM patients of different risk groups classified by the FIRST score are shown in Supplementary Table 5.

**Table 2:** Grouping and infection of the FIRST score, GEM-PETHEMA score and IRMM score.

Patients infected with the specified number (*n*)	Grade 0–2 infections	Grade 3–5 infections	*p* Value
**FIRST score**			
Low-risk (*n* = 124)	92 (74.19%)	32 (25.81%)	< 0.001
High-risk (*n* = 182)	91 (50.00%)	91 (50.00%)	
**GEM-PETHEMA score**			
Low-risk (*n* = 167)	107 (64.07%)	60 (35.93%)	0.045
Moderate-risk (*n* = 109)	64 (58.72%)	45 (41.28%)	
High-risk (*n* = 30)	12 (40.00%)	18 (60.00%)	
**IRMM score**			
Low-risk (*n* = 80)	64 (80.00%)	16 (20.00%)	< 0.001
Moderate-risk (*n* = 128)	72 (56.25%)	56 (43.75%)	
High-risk (*n* = 98)	47 (47.96%)	51 (52.04%)	

### 3.4. Evaluation of GEM-PETHEMA score

Patients were divided based on the GEM-PETHEMA score into a low-risk group (167, 54.58%), a moderate-risk group (109, 35.62%), and a high-risk group (30, 9.80%). The probability of early grade ≥ 3 infection in different stratifications showed statistically significant differences (35.93% vs. 41.28% vs. 60.00%, *χ*^2^ = 6.214, *p* = 0.045). The ORs (95% CI) for the high-risk/low-risk group, moderate-risk/low-risk group, and high-risk + moderate-risk/low-risk group were 2.675 (1.207–5.929), 1.254 (0.764–2.058), and 1.478 (0.933–2.341; *p* = 0.013, *p* = 0.370, and *p* = 0.095; Table 2). The clinical characteristics of MM patients of different risk groups classified by GEM-PETHEMA score are shown in Supplementary Table 6.

### 3.5. Evaluation of IRMM score

Patients were divided based on the IRMM score into a low-risk group (80, 26.14%), a moderate-risk group (128, 41.83%), and a high-risk group (98, 32.03%). The probability of early grade ≥ 3 infection in different stratifications showed a statistically significant difference (20.00% vs. 43.75% vs. 52.04%, *χ^2^* = 19.966, *p* < 0.001). The ORs (95% CI) for the high-risk/low-risk group, moderate-risk/low-risk group, and high-risk + moderate-risk/low-risk group were 4.340 (2.207–8.534), 3.111 (1.625–5.957), 3.597 (1.960–6.599; *p* < 0.001, *p* < 0.001, and *p* < 0.001; Table 2). The clinical characteristics of MM patients of different risk groups classified by IRMM score are shown in Supplementary Table 7.

### 3.6. Cumulative incidence of first grade ≥ 3 infection analysis of the predictive model

**Figure 3:**
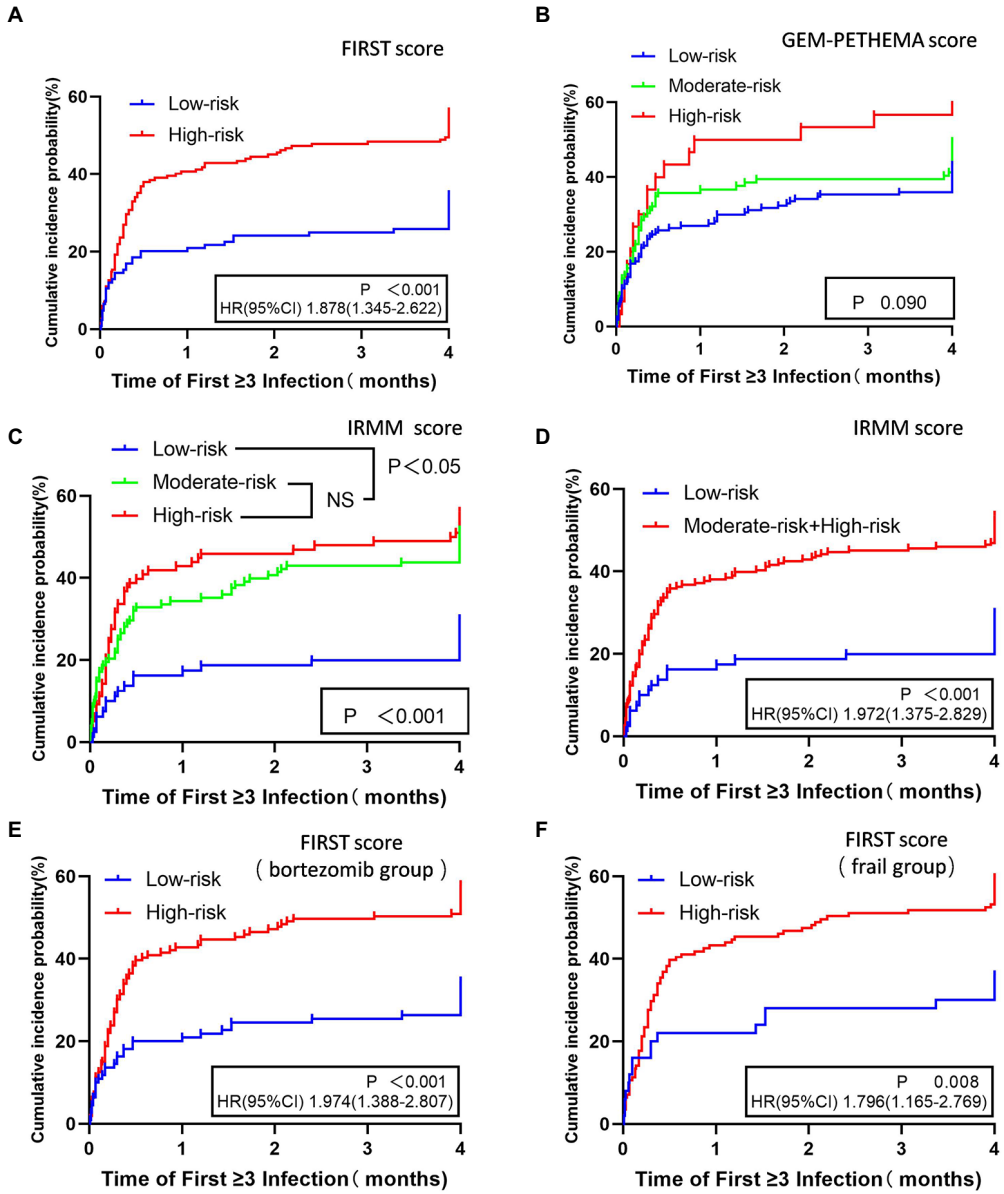
Time to first grade ≥ 3 infection in the first 4 months for different stratifications in three models. FIRST score **(A)**, GEM-PETHEMA score **(B)**, IRMM score **(C,D)**, patients treated with bortezomib scored by FIRST score **(E)**, and frail patients scored by FIRST score **(F)**. NS No statistical significance, HR Hazard ratio, CI 95% confidence interval.

The time to first infection analysis suggested that patients in the high-risk group had a higher likelihood of an early grade ≥ 3 infection in the first 4 months compared to patients in the low-risk group by FIRST score (*p* < 0.001; Figure 3A). By GEM-PETHEMA score, no statistically significant differences in the probability of early grade ≥ 3 infection were observed between the low-risk, moderate-risk, and high-risk groups (*p* = 0.090; Figure 3B). By IRMM score, statistical differences existed in the probability of early grade ≥ 3 infection between the low-risk, moderate-risk, and high-risk groups (*p* < 0.001). The high-risk and moderate-risk groups were statistically different than the low-risk group (*p* < 0.001 and *p* < 0.001, respectively). However, the difference between the high-and moderate-risk groups was not significant (*p* = 0.437; Figure 3C). For comparison with the low-risk group, the IRMM score high-and moderate-risk groups were combined into one group. This showed that the high-risk and moderate-risk groups were more likely than the low-risk group to have early grade ≥ 3 infections (*p* < 0.001; Figure 3D).

### 3.7. Comparison of prediction performance of three models

**Figure 4:**
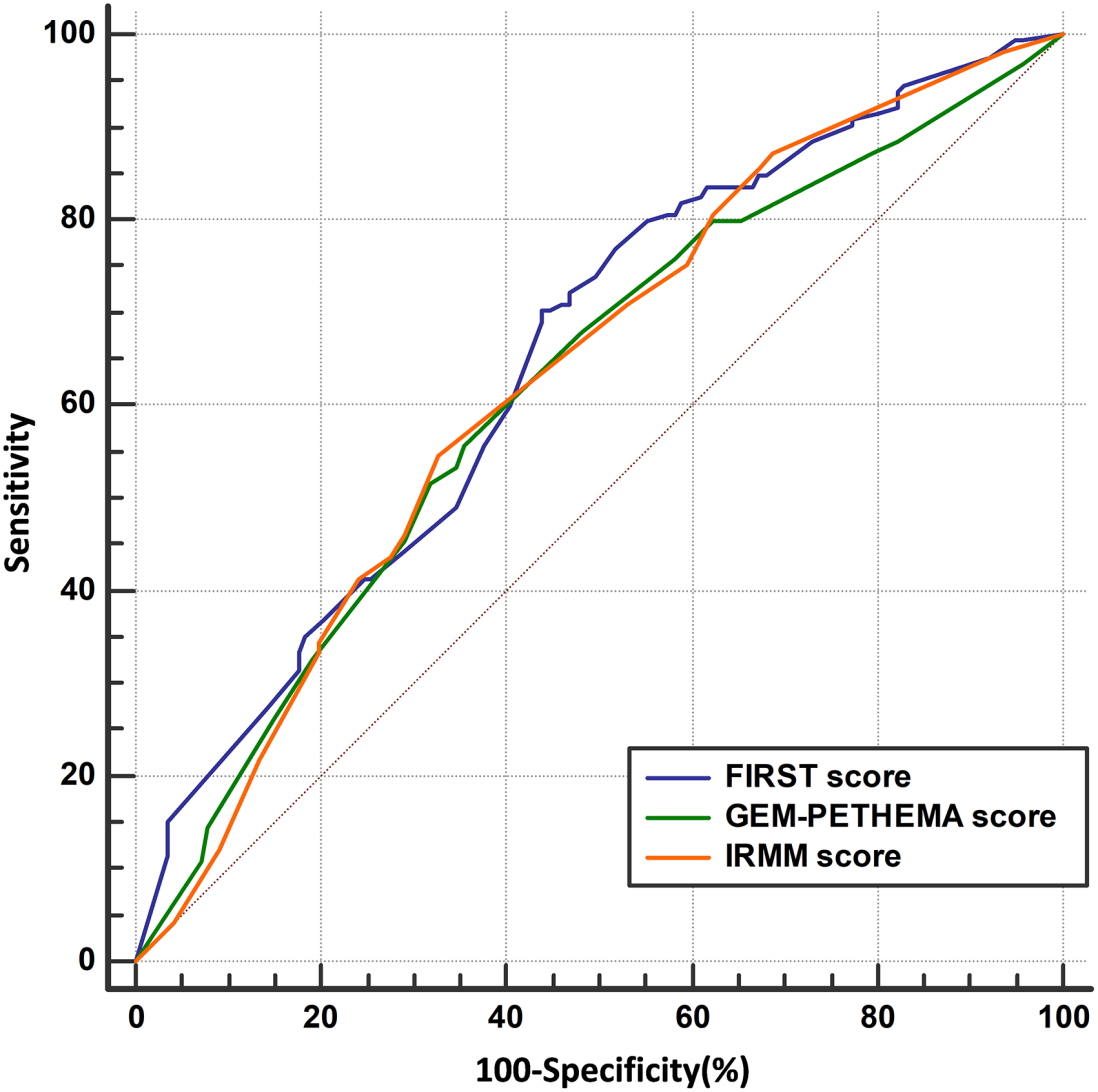
ROC curve analysis results of three models. ROC curve of FIRST score (AUC = 0.650), GEM-PETHEMA score (AUC = 0.619), and IRMM score (AUC = 0.630).

The AUC was used to compare the predictive performance between models using Med Calc software to plot the ROC, and the DeLong et al. method was used to analyze the differences in AUC and evaluate the prediction performance of the FIRST score, GEM-PETHEMA score, and IRMM score, as detailed in Figure 4 and Table 3. According to calibration plots and the decision curve analysis for the probability of infection, the difference in the predictive performance of the three models was not significant (Supplement Figures 1, 2). The FIRST score had the maximum AUC and was more discriminating for infection, although the AUCs of the three models were not statistically different. The FIRST score is also easier to implement. Hence, subgroup analysis for the FIRST score was carried out.

**Table 3:** Comparison of prediction performance in three models.

Evaluation Index	FIRST score	GEM-PETHEMA score	IRMM score
Sensitivity	0.703	0.558	0.545
Specificity	0.560	0.645	0.674
Youden index	0.263	0.203	0.219
AUC (95%CI)	0.650 (0.593–0.703)	0.619 (0.562–0.674)	0.630 (0.573–0.684)
Delong test	FIRST score vs. GEM-PETHEMA score	GEM-PETHEMA score vs. IRMM score	IRMM score vs. FIRST score
*Z* value	1.041	0.353	0.881
*p* Value	0.298	0.724	0.379

AUC, area under curve; 95%CI, 95%confidence interval.

### 3.8. Infection of different risk groups by FIRST score in bortezomib-based treatment protocols

Of the 306 patients, 269 (87.91%) were treated with the bortezomib-based regimen and 37 (12.09%) with the non-bortezomib-based regimen. Based on the FIRST score, patients treated with the bortezomib-based regimen (269 cases) were divided into a low-risk group (110, 40.89%) and a high-risk group (159, 59.11%). The high-risk group had a significantly higher probability of early grade ≥ 3 infection than the low-risk group (50.94% vs. 26.36%, *χ*^2^ = 16.252, *p* < 0.001). The OR (95% CI) for the high-risk group/low-risk group was 2.901 (1.714–4.908; Table 4). The clinical characteristics of patients treated with bortezomib-based therapy in different risk groups classified by the FIRST score are shown in Supplementary Table 8.

**Table 4:** Grouping and infection of patients treated with bortezomib-based therapy and frail patients in the FIRST score.

Patients infected with the specified number (*n*)	Grade 0–2 infections	Grade 3–5 infections	*p* Value
**Patients treated with bortezomib based therapy**			
Low-risk (*n* = 110)	81 (73.64%)	29 (26.36%)	< 0.001
High-risk (*n* = 159)	78 (49.06%)	81 (50.94%)	
**Frail patients**			
Low-risk (*n* = 50)	35 (70.00%)	15 (30.00%)	0.005
High-risk (*n* = 141)	65 (46.10%)	76 (53.90%)	

### 3.9. Infection of different risk groups by FIRST score in frail patients

Of 306 patients, 191 (62.42%) patients were in the frail group, and 115 (37.58%) were in the nonfrail group. According to the FIRST score, frail patients were divided into a low-risk group (50, 26.18%) and a high-risk group (141, 73.82%). The probability of early grade ≥ 3 infection was significantly higher in the high-risk group than in the low-risk group (53.90% vs. 30.00%, *χ*^2^ = 8.453, *p* = 0.005). The OR (95% CI) for the high-risk group/low-risk group was 2.728 (1.369–5.437; Table 4). The clinical characteristics of frail patients of different risk groups classified by the FIRST score are shown in Supplementary Table 9.

Analysis of the predictive FIRST score for the cumulative incidence of first grade ≥ 3 infection in patients treated with bortezomib based therapy and frail patients showed that patients in the high-risk group had higher probability of early grade ≥ 3 infections compared with the low-risk group (*p* < 0.001, *p* = 0.008; Figures 3E,F).

## 4. Discussion

In the present study, we first validated three grade ≥ 3 infection prediction models (FIRST score, GEM-PETHEMA score, and IRMM score) in a real-world population of patients with NDMM (mostly frail). According to our studies, the FIRST score and IRMM score could accurately predict grade ≥ 3 infection. However, the FIRST score, which comprises ECOG, β2-microglobulin, hemoglobin, and lactate dehydrogenase, is a simple and valuable infection classificatory system for patients with NDMM that could be employed in regular clinical use. The FIRST score is also suitable for frail patients and those taking bortezomib-based therapy.

Our results show that 66.01% (202/306) of NDMM patients reported infections within the first 4 months from the initiation of treatment, and 40.20% (123/306) ≥ 3 TE infections. The total number of any grade infections in this study was higher than the results of other series (37.3–61.8%; Rosiñol et al., 2019; Voorhees et al., 2020). It was also higher when compared with grade ≥ 3 infection in prior trials (11.9–20.37%; Dumontet et al., 2018; Encinas et al., 2022; Shang et al., 2022). Most of the participants in our research were frail patients, and we do not perform antibacterial prophylaxis for patients, so that may be the reason for the high rate of infection, especially for ≥ 3 TE infections. It makes sense to develop infection risk models. The most common pathogens in infections were Gram-negative bacteria, as in previous studies (Teh et al., 2017). As shown in previous similar studies, the most common site of infection was the respiratory tract (Teh et al., 2015; Zahid et al., 2019). The mortality associated with infection in the first 4 months was low (2.61%), which is consistent with the results of other series (1.34–5.86%; Augustson et al., 2005; Jung et al., 2016; Huang et al., 2017; Terebelo et al., 2017).

Besides the uniform clinical trials, a robust risk stratification system should work effectively in a wide range of clinical circumstances. This study demonstrates that the FIRST score can predict grade ≥ 3 early infection in the real world and shows reasonable distinction. The infection rate of the high-risk group was significantly higher than that of the low-risk group when divided by FIRST score (50.00% vs. 25.81%, *p* < 0.001). The GEM-PETHEMA score performed poorly in infection prediction and group discrimination. Based on real-world data, statistical differences existed in the incidence of infection between the high-risk group and the low-risk group (60.00% vs. 35.93%, *p* = 0.013) by GEM-PETHEMA score, but with no statistical difference between the moderate-risk group and the low-risk group (41.28% vs. 35.93%, *p* = 0.370) or between the high-risk group and the moderate-risk group (60.00% vs. 41.28%, *p* = 0.095). Furthermore, the cumulative incidence of infection did not differ statistically between the three GEM-PETHEMA score groups (*p* = 0.090). This indicates that the GEM-PETHEMA score does not appear to have effective discrimination for grade ≥ 3 infection in the real world. When the GEM-PETHEMA score research group tested the FIRST score using their data, they found no significant association with a higher risk of early severe infection between the high-risk and low-risk groups (*p* = 0.347; Encinas et al., 2022). However, our study results show that the FIRST score had a relatively high predictive value compared to the GEM-PETHEMA score. Differences in the patients included may also be the reason. The FIRST score’s patients are comprised of transplant-ineligible candidates. In the GEM-PETHEMA score, patients who also includes transplant-eligible candidates. In contrast, the patients in our study were similar to the patients in the FIRST score study, and the majority of them were transplant-ineligible candidates. However, the exact reason remains unclear, and this difference of results should be answered in future studies. The IRMM score is based on real-world data. The findings of this study indicate that the IRMM score had an elevated predictive value. When we inspected the high-risk, moderate-risk, and low-risk groups by IRMM score, we found significant differences between the high-risk and low-risk groups (*p* < 0.001) and between the moderate-risk and low-risk groups (*p* < 0.001). However, the differentiation between the high-risk and moderate-risk groups was unclear (*p* = 0.437).

This study attempted to further analyze these three models as to which may have a better ability to distinguish grade ≥ 3 infection in NDMM patients using ROC curves and the Youden index. However, from the validation results, the three prediction models were not much different. The probability of grade ≥ 3 infection in different infection groups was compared (*p* < 0.05), as well as the sensitivity and specificity of the three models (*p* > 0.05). However, the FIRST score model was only slightly better (AUC: 0.650 vs. 0.619 vs. 0.630). In addition, the cumulative infection rate by the FIRST and IRMM scores in the first 4 months was significantly statistically different, whereas the GEM-PETHEMA score showed no significant difference. Additionally, considering the prediction indicators and calculation process, the calculation of the IRMM score is relatively complex, whereas the calculation of FIRST and GEM-PETHEMA scores is relatively simple. Combining validated results and computational processes, we believe that the FIRST score will be more beneficial in clinical practice.

However, notably, induction regimens for developing the FIRST score did include non-proteasome-based regimens. In the real world, especially in China, most patients are treated with bortezomib-based regimens (Chinese Hematology Association, 2020; Zhou et al., 2022). Our results show that the FIRST score can be well-verified in real-world data. In addition, in the real world, the proportion of frail NDMM patients who are not transplanted is high (Antoine-Pepeljugoski and Braunstein, 2019; Goel et al., 2022; Medhekar et al., 2022; Yao et al., 2022). This study further explores the application value in frail patients, and the results show that in frail patients, the FIRST score can still carry out stratification.

Monitoring high-risk patients and taking active relevant preventive measures may be significant. The indications for antimicrobial prophylaxis are controversial. A randomized phase III URCC/ECOG study showed that the use of prophylactic antibiotics during the first 2 months of treatment did not reduce the incidence of severe infection (Vesole et al., 2012). In contrast, recent TEAMM results have shown that prophylactic use of levofloxacin in active myeloma treatment during the first 12 weeks of therapy significantly reduced febrile episodes and deaths compared to placebo (Kim et al., 2022). Nevertheless, prophylactic antibiotics are not recommended for all patients (Girmenia et al., 2019). The IMWG infection consensus guidelines and recommendation suggests that antimicrobial prophylaxis should be targeted at people at high or moderate risk of MM infection (Raje et al., 2022). Following the findings of this study, the FIRST score can better monitor high-risk patients. The FIRST score application facilitates infection prevention or adjustment of the intensity of induction therapy to reduce the incidence of infection.

Our study has several limitations. First, despite multicenter data collection, the sample size was relatively small. The nature of retrospective design narrows the conclusion, so biases were unpreventable. Second, the regimen containing daratumumab has been recommended for the first-line treatment of NDMM (Lonial et al., 2016). However, because this drug is not yet included in medical insurance reimbursement in China, few patients can use the regimen containing daratumumab for first-line treatment. Studies have reported that the incidence of infection is relatively high in the treatment of monoclonal antibody–based regimens, and these three models must be further verified in the regimen containing daratumumab. Third, based on the background of the COVID-19 pandemic, COVID-19 infection in NDMM, infection rates, and risk factors need to be investigated further, which can be done in follow-up studies.

In conclusion, our study confirms that the FIRST score is a simple and robust infection stratification tool for patients with NDMM, including frail patients and those being treated with bortezomib-based therapy. It can discriminate patients with grade ≥ 3 infection, help to guide infection prevention, and improve outcomes of patients with NDMM.

## Data availability statement

The original contributions presented in the study are included in the article/Supplementary material, further inquiries can be directed to the corresponding authors.

## Ethics statement

The studies involving human participants were reviewed and approved by Shanxi Bethune hospital ethics committee. Written informed consent for participation was waived for this study.

## Author contributions

WT and JW designed and supervised the work, interpreted the results, and approved the final version. XL, WL, LZ, LY, QY, JZ, SH, and XC collected clinical data. XL and XC analyzed data. XL, WL, WT, and JW wrote and revised the manuscript. All authors contributed to the article and approved the submitted version.

## Funding

This research did not receive any specific grant from funding agencies in the public, commercial, or not-for-profit sectors.

## Conflict of interest

The authors declare that the research was conducted in the absence of any commercial or financial relationships that could be construed as a potential conflict of interest.

## Publisher’s note

All claims expressed in this article are solely those of the authors and do not necessarily represent those of their affiliated organizations, or those of the publisher, the editors and the reviewers. Any product that may be evaluated in this article, or claim that may be made by its manufacturer, is not guaranteed or endorsed by the publisher.
