# Supplementary material for: Eastern Cooperative Oncology Group, β2-microglobulin, hemoglobin, and lactate dehydrogenase can predict early grade ≥ 3 infection in patients with newly diagnosed multiple myeloma: A real-world multicenter study
Source: Front Microbiol. 2023 Jan 27;14:1114972. doi: 10.3389/fmicb.2023.1114972 (PMC9911534; doi:10.3389/fmicb.2023.1114972)
Supplement: Supplementary file 1 [file Data_Sheet_1.docx]

Supplementary Material

**This file includes:**

Figures S1 to S2

Tables S1 to S7

**CATALOGUE**

[Supplementary Figures 1](#_Toc7789)

[Supplementary Tables 3](#_Toc11958)

[Supplementary Table 1. The values of the three predictive models and the grouping criteria 3](#_Toc16372)

[Supplementary Table 2. Comparison of baseline characteristics in patients with NDMM 5](#_Toc1052)

[Supplementary Table 3. Infection status of different induction treatment 7](#_Toc21867)

[Supplementary Table 4. Classification and constituent ratios (%) of pathogens in patients with NDMM 9](#_Toc14295)

[Supplementary Table 5. Clinical Characteristics of Patients with NDMM of different risk groups classified by FIRST score 11](#_Toc15995)

[Supplementary Table 6. Clinical characteristics of NDMM patients of different risk groups classified by GEM-PETHEMA score 14](#_Toc19970)

[Supplementary Table 7. Clinical characteristics of NDMM patients of different risk groups classified by IRMM score 17](#_Toc28036)

[Supplementary Table 8. Clinical characteristics of patients treated with bortezomib based therapy of different risk groups classified by FIRST score 20](#_Toc15214)

[Supplementary Table 9. Clinical characteristics of frail patients of different risk groups classified by FIRST score 23](#_Toc18517)

# Supplementary Figures

**
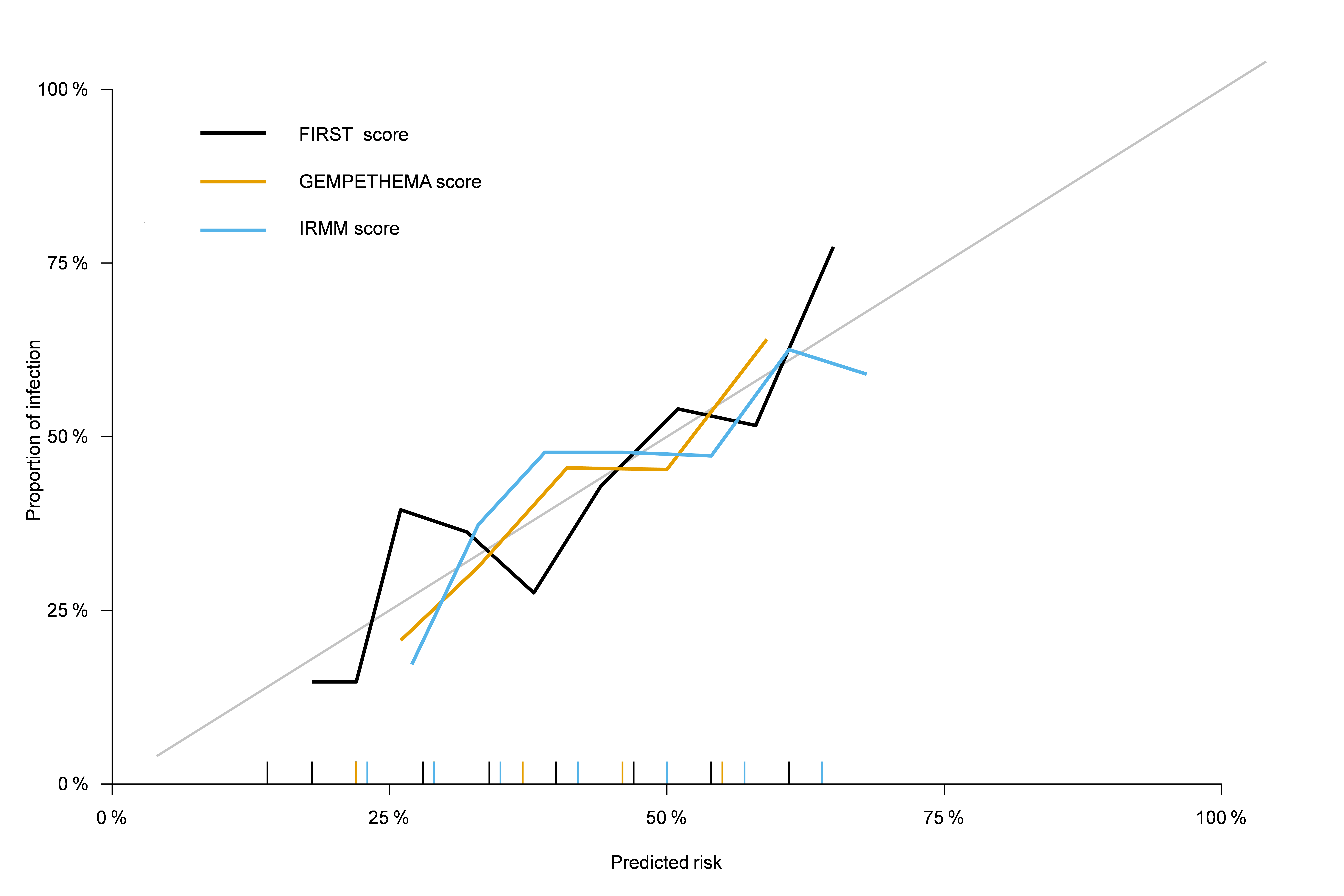
**

**Supplementary Figure 1.** **Calibration curve of FIRST score, GEM-PETHEMA score, and IRMM score**

The fitness of the predicted probabilities of the three models to their actual results was evaluated. The probability of three models is represented by the x-axis, while the actual probability of three models is represented by the y-axis. The diagonal line represents the ideal model's best estimates.

**
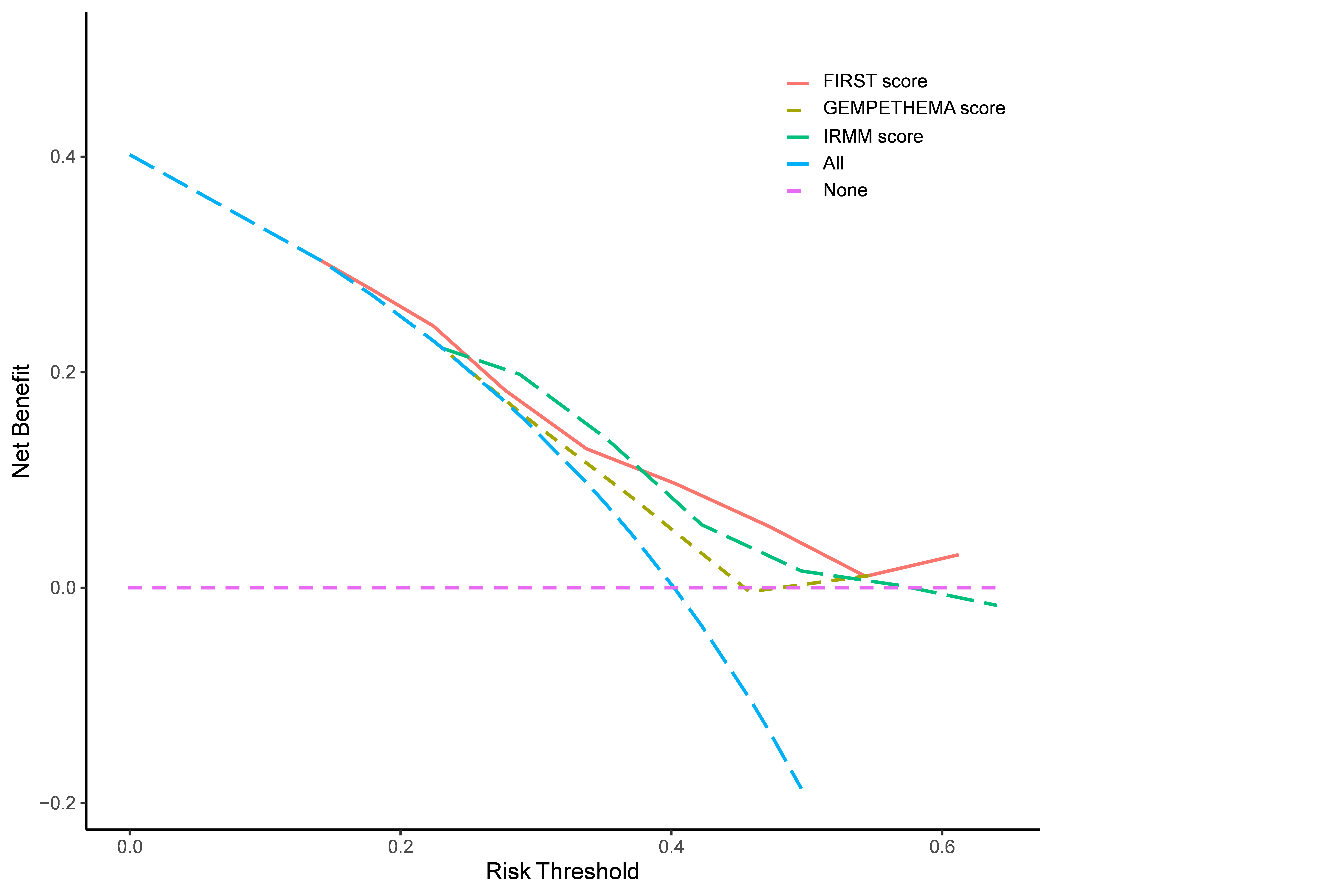
Supplementary Figure 2.** **Decision Curve Analysis of FIRST score, GEM-PETHEMA score, and IRMM score**

The x-axis demonstrates the threshold probability, as the y-axis demonstrates net benefit.The decision curve is generated by plotting net benefit as a function of threshold level.This chart illustrates that the FIRST score, GEM-PETHEMA score, and IRMM score both lead to very similar net benefit values over a wide range of threshold levels and that none of the models alone can lead to similarly high net benefit values.

# Supplementary Tables

**Supplementary Table 1. The values of the three predictive models and the grouping criteria**

| Predictive models | Factor | Points | Grouping criteria |
| --- | --- | --- | --- |
| FIRST score | β2-MG ≤3 mg/L | -2 | -3 - 1 points,low-risk  2-5 points,high-risk |
|  | ECOG ＝0 | -1 |  |
|  | ECOG ≥ 2 | 1 |  |
|  | hemoglobin ≤ 110 g/L | 1 |  |
|  | LDH ≥ 200 U/L | 1 |  |
|  | β2-MG ≥ 6 mg/L | 2 |  |
|  |  |  |  |
| GEM-PETHEMA score | serum albumin ≤ 30 g/l | 1 | 0-2 points,low-risk  3 points,moderate risk  4 points,high-risk |
|  | ECOG > 1 | 1 |  |
|  | male | 1 |  |
|  | non-IgA MM | 1 |  |
|  |  |  |  |
| IRMM score | hemoglobin was ＜35 g/L of the lower limit of the normal range | 1 | 0-1 point,low-risk  2-3 points,moderate risk  4-6 points,high-risk |
|  | globulin ≥2.1 times the upper limit of the normal range | 1 |  |
|  | ECOG ≥ 2 | 2 |  |
|  | β2-MG≥ 6 mg/L | 2 |  |

ECOG Eastern Cooperative Oncology Group Performance Status, β2-MG Serum β2-microglobulin, LDH Lactate dehydrogenase, IgA Immunoglobulin A.

**Supplementary Table 2. Comparison of baseline characteristics in patients with NDMM**

| Patient characteristics | Uninfected（n=104） | Grade 1-2 infections（n=79） | Grade 3-5 infections（n=123） | *P value* |
| --- | --- | --- | --- | --- |
| Age(years)  ≥65  ＜65 | 46(44.23%)  58(55.77%) | 45(56.96%)  34(43.04%) | 61(49.59%)  62(50.41%) | 0.227 |
| Sex  Male  Female | 58(55.77%)  46(44.23%) | 47(59.49%)  32(40.51%) | 67(54.47%)  56(45.53%) | 0.776 |
| MM subtype  IgA  Non-IgA | 25(24.04%)  79(75.96%) | 20(25.32%)  59(74.68%) | 25(20.33%)  98(79.67%) | 0.693 |
| ISS stage  Ⅰ-Ⅱ  Ⅲ | 58(55.77%)  46(44.23%) | 38(48.10%)  41(51.90%) | 46(37.40%)  77(62.60%) | 0.025 |
| DS stage  Ⅰ-Ⅱ  Ⅲ | 17(16.35%)  87(83.65%) | 10(12.66%)  69(87.34%) | 14(11.38%)  109(88.62%) | 0.533 |
| ECOG  0  1  2-4 | 22(21.15%)  39(37.50%)  43(41.35%) | 3(3.80%)  26(32.91%)  50(63.29%) | 5(4.06%)  36(29.27%)  82(66.67%) | ＜0.001 |
| Frailty assessment  Frail  Nonfrail | 46(44.23%)  58(55.77%) | 54(68.35%)  25(31.65%) | 91(73.98%)  32(26.02%) | ＜0.001 |
| Hemoglobin (g/L) | 97.58±26.53 | 92.88±24.41 | 86.77±20.38 | 0.003 |
| Platelet(×10^9^/L) | 168.29±74.06 | 178.59±92.96 | 164.95±88.90 | 0.532 |
| WBC(×10^9^/L) | 4.65±2.07 | 4.99±3.02 | 5.83±3.21 | 0.006 |
| Serum β2-microglobulin(mg/L) | 5.73±5.26 | 7.19±5.37 | 8.86±7.14 | 0.001 |
| Lactate dehydrogenase(U/L) | 166.07±89.95 | 179.80±65.22 | 215.37±164.39 | 0.006 |
| Albumin(g/L) | 32.65±6.80 | 32.34±7.20 | 30.77±7.45 | 0.109 |
| Globulin(g/L) | 43.20±27.14 | 50.57±31.30 | 52.47±28.82 | 0.047 |

NDMM Newly diagnosed multiple myeloma, IgA Immunoglobulin A, ISS Stage International Staging System Stage, DS Stage Durie-Salmon stage, ECOG Eastern Cooperative Oncology Group Performance Status,WBC White blood cells, The severity of infection was evaluated using the Common Terminology Criteria for Adverse Events v5.0 (CTCAE) published by the National Cancer Institute of the National Institutes of Health, U.S. Department of Health and Human Services.

**Supplementary Table 3. Infection status of different induction treatment**

| Induction treatment | Uninfected（n=104） | Grade 1-2 infections（n=79） | Grade 3-5 infections（n=123） | *P value* |
| --- | --- | --- | --- | --- |
| Bortezomib-based regimen(n=269)  Triplet regimen(n=172)^a^  Doublet regimen(n=91)^b^  Others(n=6)^c^ | 63  27  2 | 49  18  0 | 60  46  4 | 0.068 |
| Non-bortezomib-based regimen (n=37)  Triplet regimen(n=14)^d^  Doublet regimen(n=16)^e^  Others(n=7)^f^ | 6  5  1 | 5  5  2 | 3  6  4 | 0.556 |

a: Bortezomib+ Cyclophosphamide+ Dexamethasone(VCD), Bortezomib+ Lenalidomide+ Dexamethasone(VRD), Bortezomib +Pomalidomide+Dexamethasone(VPD), Bortezomib+Thalidomide+Dexamethasone(VTD);

b: Bortezomib+ Dexamethasone(VD);

c:Bortezomib(V),Bortezomib+Thalidomide+Dexamethasone+Cyclophosphamide(VTDC),Bortezomib+ Dexamethasone+Etoposide+Cyclophosphamide+Cisplatin(VDECP);

d: Lenalidomide+ Cyclophosphamide +Dexamethasone(RCD), Thalidomide+ Cyclophosphamide+ Dexamethasone(TCD), Ixazomib+ Lenalidomide+ Dexamethasone(IRD),Ixazomib+cyclophosphamide+ Dexamethasone(ICD);

e:Lenalidomide + Dexamethasone(RD),Ixazomib+ Dexamethasone(ID), Thalidomide+ Dexamethasone(TD);

f:Doxorubicin+Vincristine+Dexamethasone(DVD), Lenalidomide(R).

The severity of infection was evaluated using the Common Terminology Criteria for Adverse Events v5.0 (CTCAE) published by the National Cancer Institute of the National Institutes of Health, U.S. Department of Health and Human Services.

**Supplementary Table 4. Classification and constituent ratios (%) of pathogens in patients with NDMM**

| **Pathogens** | **N=88** | **Ratio（%）** |
| --- | --- | --- |
| Bacteria | 59 | 67.05% |
| Gram negative bacteria | 42 | 47.73% |
| *Escherichia coli* | 14 | 15.91% |
| *Klebsiella pneumoniae* | 11 | 12.50% |
| *Stenotrophomonas maltophilia* | 7 | 7.95% |
| *Acinetobacter baumannii* | 4 | 4.55% |
| *Pseudomonas aeruginosa* | 2 | 2.27% |
| *Salmonella enteritidis* | 1 | 1.14% |
| *Haemophilus influenzae* | 1 | 1.14% |
| *Serratia marcescens* | 1 | 1.14% |
| *Enterobacter hormaechei* | 1 | 1.14% |
| Gram positive bacteria | 17 | 19.32% |
| *Enterococcus faecium* | 5 | 5.68% |
| *Staphylococcus aureus* | 3 | 3.41% |
| *Clostridium* | 3 | 3.41% |
| *Staphylococcus haemolyticus* | 2 | 2.27% |
| *Staphylococcus capitis* | 1 | 1.14% |
| *Staphylococcus epidermidis* | 1 | 1.14% |
| *Listeria monocytogenes* | 1 | 1.14% |
| *Streptococcus gallolyticus* | 1 | 1.14% |
| *Virus* | 20 | 22.73% |
| *Herpes simplex virus* | 9 | 10.23% |
| *Varicella-zoster virus* | 7 | 7.95% |
| *Epstein-Barr virus* | 2 | 2.27% |
| *Respiratory syncytial virus* | 1 | 1.14% |
| *Hepatitis C virus* | 1 | 1.14% |
| *Fungus* | 9 | 10.23% |
| *Candida albicans* | 6 | 6.82% |
| *Aspergillus niger* | 2 | 2.27% |
| *Aspergillus fumigatus* | 1 | 1.14% |

NDMM Newly diagnosed multiple myeloma

**Supplementary Table 5. Clinical Characteristics of Patients with NDMM of different risk groups classified by FIRST score**

| Clinical Parameters | low-risk（n=124） | high-risk（n=182） | *P* value |
| --- | --- | --- | --- |
| ECOG  0  1  2-4 | 21(16.94%)  60(48.39%)  43(34.67%) | 9(4.95%)  41(22.53%)  132(72.52%) | ＜0.001 |
| Hb  Hb≤90g/L  90g/L＜Hb＜110 g/L  Hb≥110g/L | 33(26.62%)  30(24.19%)  61(49.19%) | 126(69.23%)  45(24.73%)  11(6.04%) | ＜0.001 |
| β2-MG  β2-MG≤3mg/L  3mg/L＜β2-MG＜6mg/L  β2-MG≥6mg/L | 82(66.13%)  42(33.87%)  0(0.00%) | 0(0.00%)  53(29.12%)  129(70.88%) | ＜0.001 |
| LDH  LDH ＜200 U/L  LDH ≥200 U/L | 108(87.10%)  16(12.90%) | 115(63.19%)  67(36.81%) | ＜0.001 |
| Age  ≥65  ＜65 | 49(39.52%)  75(60.48%) | 103(56.59%)  79(43.41%) | 0.004 |
| Sex  Male  Female | 69(55.65%)  55(44.35%) | 103(56.59%)  79(43.41%) | 0.907 |
| MM subtype  IgA  Non-IgA | 28(22.58%)  96(77.42%) | 42(23.08%)  140(76.92%) | 1.000 |
| ISS stage  Ⅰ-Ⅱ  Ⅲ | 95(76.61%)  29(23.39%) | 47(25.82%)  135(74.18%) | ＜0.001 |
| Frailty assessment  Frail  Nonfrail | 50(40.32%)  74(59.68%) | 141(77.47%)  41(22.53%) | ＜0.001 |
| Albumin  ≤30g/L  ＞30g/L | 42(33.87%)  82(66.13%) | 89(48.90%)  93(51.10%) | 0.010 |
| Treatment protocol  Bortezomib-based  Non-bortezomib-based | 110(88.71%)  14(11.29%) | 159(87.36%)  23(12.64%) | 0.859 |
| Infections（%） | 63/124（50.81%） | 139/182（76.37%） |  |
| Grade ≥3 infections（%） | 32/124(25.81%) | 91/182(50.00%) | ＜0.001 |

NDMM Newly diagnosed multiple myeloma, ECOG Eastern Cooperative Oncology Group Performance Status,Hb Hemoglobin, β2-MG β2microglobulin, LDH Lactate dehydrogenase, MM Multiple myeloma, IgA Immunoglobulin A, ISS Stage International Staging System Stage, The severity of infection was evaluated using the Common Terminology Criteria for Adverse Events v5.0 (CTCAE) published by the National Cancer Institute of the National Institutes of Health, U.S. Department of Health and Human Services.

**Supplementary Table 6. Clinical characteristics of NDMM patients of different risk groups classified by GEM-PETHEMA score**

| Clinical Parameters | low-risk（n=167） | moderate-risk（n=109） | high-risk（n=30） | *P* value |
| --- | --- | --- | --- | --- |
| Albumin  ≤30g/L  ＞30g/L | 30(17.96%)  137(82.04%) | 71(65.14%)  38(34.86%) | 30(100.00%)  0(0.00%) | ＜0.001 |
| ECOG  0  1-4 | 25(14.97%)  142(85.03%) | 5(4.59%)  104(95.41%) | 0(0.00%)  30(100.00%) | 0.003 |
| Sex  Male  Female | 61(36.53%)  106(63.47%) | 81(74.31%)  28(25.69%) | 30(100.00%)  0(0.00%) | ＜0.001 |
| MM subtype  IgA  Non-IgA | 53(31.74%)  114(68.26%) | 17(15.60%)  92(84.40%) | 0(0.00%)  30(100.00%) | ＜0.001 |
| Age  ≥65  ＜65 | 69(41.32%)  98(58.68%) | 57(52.29%)  52(47.71%) | 26(86.67%)  4(13.33%) | ＜0.001 |
| ISS stage  Ⅰ-Ⅱ  Ⅲ | 81(48.50%)  86(51.50%) | 51(46.79%)  58(53.21%) | 10(33.33%)  20(66.67%) | 0.307 |
| Frailty assessment  Frail  Nonfrail | 76(45.51%)  91(54.49%) | 85(77.98%)  24(22.02%) | 30(100.00%)  0(0.00%) | ＜0.001 |
| Hb  Hb≤90g/L  90g/L＜Hb＜110 g/L  Hb≥110g/L | 80(47.91%)  40(23.95%)  47(28.14%) | 58(53.21%)  30(27.52%)  21(19.27%) | 21(70.00%)  5(16.67%)  4(13.33%) | 0.121 |
| β2-MG  β2-MG≤3mg/L  3mg/L＜β2-MG＜6mg/L  β2-MG≥6mg/L | 56(33.53%)  50(29.94%)  61(36.53%) | 23(21.10%)  34(31.19%)  52(47.71%) | 3(10.00%)  11(36.67%)  16(53.33%) | 0.031 |
| LDH  LDH ＜200 U/L  LDH ≥200 U/L | 126(75.45%)  41(24.55%) | 75(68.81%)  34(31.19%) | 22(73.33%)  8(26.67%) | 0.478 |
| Treatment protocol  Bortezomib-based  Non-bortezomib-based | 152(91.02%)  15(8.98%) | 92(84.40%)  17(15.60%) | 25(83.33%)  5(16.67%) | 0.185 |
| Infections（%） | 99/167（59.28%） | 77/109（70.64%） | 26/30（86.67%） |  |
| Grade ≥3 infections（%） | 60/167(35.93%) | 45/109（41.28%） | 18/30（60.00%） | 0.045 |

NDMM Newly diagnosed multiple myeloma, ECOG Eastern Cooperative Oncology Group Performance Status, MM Multiple myeloma, IgA Immunoglobulin A, ISS Stage International Staging System Stage, Hb Hemoglobin, β2-MG β2microglobulin, LDH Lactate dehydrogenase, The severity of infection was evaluated using the Common Terminology Criteria for Adverse Events v5.0 (CTCAE) published by the National Cancer Institute of the National Institutes of Health, U.S. Department of Health and Human Services.

**Supplementary Table 7. Clinical characteristics of NDMM patients of different risk groups classified by IRMM score**

| Clinical Parameters | low-risk（n=80） | moderate-risk（n=128） | high-risk（n=98） | *P* value |
| --- | --- | --- | --- | --- |
| ECOG  0-1  2-4 | 80(100.00%)  0(0.00%) | 42(32.81%)  86(67.19%) | 9(9.18%)  89(90.82%) | ＜0.001 |
| Hb  ＜35 g/L of the lower limit of the normal range  ≥35 g/L of the lower limit of the normal range | 12(15.00%)  68(85.00%) | 34(26.56%)  94(73.44%) | 63(64.29%)  35(35.71%) | ＜0.001 |
| β2-MG  β2-MG＜6mg/L  β2-MG≥6mg/L | 80(100.00%)  0(0.00%) | 91(71.09%)  37(28.91%) | 6(6.12%)  92(93.88%) | ＜0.001 |
| Glb  ≥2.1 times the upper limit of the normal range  ＜2.1 times the upper limit of the normal range | 15(18.75%)  65(81.25%) | 24(18.75%)  104(81.25%) | 48(48.98%)  50(51.02%) | ＜0.001 |
| LDH  LDH ＜200 U/L  LDH ≥200 U/L | 66(82.50%)  14(17.50%) | 94(73.44%)  34(26.56%) | 63(64.29%)  35(35.71%) | 0.024 |
| Age  ≥65  ＜65 | 25(31.25%)  55(68.75%) | 66(51.56%)  62(48.44%) | 61(62.24%)  37(37.76%) | ＜0.001 |
| Sex  Male  Female | 43(53.75%)  37(46.25%) | 72(56.25%)  56(43.75%) | 57(58.16%)  41(41.84%) | 0.840 |
| MM subtype  IgA  Non-IgA | 17(21.25%)  63(78.75%) | 31(24.22%)  97(75.78%) | 22(22.45%)  76(77.55%) | 0.878 |
| ISS stage  Ⅰ-Ⅱ  Ⅲ | 60(75.00%)  20(25.00%) | 68(53.12%)  60(46.88%) | 14(14.29%)  84(85.71%) | ＜0.001 |
| Frailty assessment  Frail  Nonfrail | 7(8.75%)  73(91.25%) | 94(73.44%)  34(26.56%) | 90(91.84%)  8(8.16%) | ＜0.001 |
| Albumin  ≤30g/L  ＞30g/L | 28(35.00%)  52(65.00%) | 41(32.03%)  87(67.97%) | 62(63.27%)  36(36.73%) | ＜0.001 |
| Treatment protocol  Bortezomib-based  Non-bortezomib-based | 73(91.25%)  7(8.75%) | 116(90.62%)  12(9.38%) | 80(81.63%)  18(18.37%) | 0.069 |
| Infections（%） | 33/80（41.25%） | 92/128（71.88%） | 77/98（78.57%） |  |
| Grade ≥3 infections（%） | 16/80（20.00%） | 56/128（43.75%） | 51/98（52.04%） | ＜0.001 |

NDMM Newly diagnosed multiple myeloma, ECOG Eastern Cooperative Oncology Group Performance Status, Hb Hemoglobin, β2-MG β2microglobulin, Glb Globulin, LDH Lactate dehydrogenase, MM Multiple myeloma, IgA Immunoglobulin A, ISS Stage International Staging System Stage, The severity of infection was evaluated using the Common Terminology Criteria for Adverse Events v5.0 (CTCAE) published by the National Cancer Institute of the National Institutes of Health, U.S. Department of Health and Human Services.

**Supplementary Table 8. Clinical characteristics of patients treated with bortezomib based therapy of different risk groups classified by FIRST score**

| Clinical Parameters | low-risk  （n=110） | high-risk  （n=159） | *P* value |
| --- | --- | --- | --- |
| ECOG  0  1  2-4 | 21(19.09%)  54(49.09%)  35(31.82%) | 8(5.03%)  38(23.90%)  113(71.07%) | ＜0.001 |
| Hb  Hb≤90g/L  90g/L＜Hb＜110  Hb≥110g/L | 29(26.36%)  30(27.27%)  51(46.37%) | 111(69.81%)  39(24.53%)  9(5.66%) | ＜0.001 |
| β2-MG  β2-MG≤3mg/L  3mg/L＜β2-MG＜6mg/L  β2-MG≥6mg/L | 70(63.64%)  40(36.36%)  0(0.00%) | 0(0.00%)  48(30.19%)  111(69.81%) | ＜0.001 |
| LDH  LDH ＜200 U/L  LDH ≥200 U/L | 96(87.27%)  14(12.73%) | 102(64.15%)  57(35.85%) | ＜0.001 |
| Age  ≥65  ＜65 | 43(39.09%)  67(60.91%) | 87(54.72%)  72(45.28%) | 0.013 |
| Sex  Male  Female | 59(53.64%)  51(46.36%) | 91(57.23%)  68(42.77%) | 0.618 |
| MM subtype  IgA  Non-IgA | 23(20.91%)  87(79.09%) | 37(23.27%)  122(76.73%) | 0.659 |
| ISS stage  Ⅰ-Ⅱ  Ⅲ | 84(76.36%)  26(23.64%) | 43(27.04%)  116(72.96%) | ＜0.001 |
| Frailty assessment  Frail  Nonfrail | 42(38.18%)  68(61.82%) | 121(76.10%)  38(23.90%) | ＜0.001 |
| Albumin  ≤30g/L  ＞30g/L | 37(33.64%)  73(66.36%) | 75(47.17%)  84(52.83%) | 0.032 |
| Infections（%） | 57/110(51.82%) | 120/159(75.47%) |  |
| Grade ≥3 infections（%） | 29/110（26.36%） | 81/159（50.94%） | ＜0.001 |

ECOG Eastern Cooperative Oncology Group Performance Status, Hb Hemoglobin, β2-MG β2microglobulin, LDH Lactate dehydrogenase, MM Multiple myeloma, IgA Immunoglobulin A, ISS Stage International Staging System Stage, The severity of infection was evaluated using the Common Terminology Criteria for Adverse Events v5.0 (CTCAE) published by the National Cancer Institute of the National Institutes of Health, U.S. Department of Health and Human Services.

**Supplementary Table 9. Clinical characteristics of frail patients of different risk groups classified by FIRST score**

| Clinical Parameters | low-risk（n=50） | high-risk（n=141） | *P* value |
| --- | --- | --- | --- |
| ECOG  0  1  2-4 | 0(0.00%)  7(14.00%)  43(86.00%) | 0(0.00%)  9(6.38%)  132(93.62%) | 0.134 |
| Hb  Hb≤90g/L  90g/L＜Hb＜110  Hb≥110g/L | 8(16.00%)  16(32.00%)  26(52.00%) | 93(65.96%)  38(26.95%)  10(7.09%) | ＜0.001 |
| β2-MG  β2-MG≤3mg/L  3mg/L＜β2-MG＜6mg/L  β2-MG≥6mg/L | 38(76.00%)  12(24.00%)  0(0.00%) | 0(0.00%)  49(34.75%)  92(65.25%) | ＜0.001 |
| LDH  LDH ＜200 U/L  LDH ≥200 U/L | 44(88.00%)  6(12.00%) | 94(66.67%)  47(33.33%) | 0.005 |
| Age  ≥65  ＜65 | 29(58.00%)  21(42.00%) | 93(65.96%)  48(34.04%) | 0.392 |
| Sex  Male  Female | 30(60.00%)  20(40.00%) | 76(53.90%)  65(46.10%) | 0.510 |
| MM subtype  IgA  Non-IgA | 13(26.00%)  37(74.00%) | 36(25.53%)  105(74.47%) | 1.000 |
| ISS stage  Ⅰ-Ⅱ  Ⅲ | 39(78.00%)  11(22.00%) | 43(30.50%)  98(69.50%) | ＜0.001 |
| Treatment protocol  Bortezomib-based  Non-bortezomib-based | 42(84.00%)  8(16.00%) | 121(85.82%)  20(14.18%) | 0.817 |
| Albumin  ≤30g/L  ＞30g/L | 16(32.00%)  34(68.00%) | 73(51.77%)  68(48.23%) | 0.021 |
| Infections（%） | 33/50（66.00%） | 112/141（79.43%） |  |
| Grade ≥3 infections（%） | 15/50（30.00%） | 76/141（53.90%） | 0.005 |

ECOG Eastern Cooperative Oncology Group Performance Status, Hb Hemoglobin, β2-MG β2microglobulin, LDH Lactate dehydrogenase, MM Multiple myeloma, IgA Immunoglobulin A, ISS Stage International Staging System Stage, The severity of infection was evaluated using the Common Terminology Criteria for Adverse Events v5.0 (CTCAE) published by the National Cancer Institute of the National Institutes of Health, U.S. Department of Health and Human Services.
